# The efficacy of a health-related quality-of-life intervention during 48 weeks of biologic treatment of patients with moderate to severe psoriasis: study protocol for a multicenter randomized controlled trial

**DOI:** 10.1186/1745-6215-13-236

**Published:** 2012-12-08

**Authors:** Cecilia AC Prinsen, Phyllis I Spuls, Mirjam AG Sprangers, Menno A de Rie, Catharina M Legierse, John de Korte

**Affiliations:** 1 Academic Medical Center, Department of Dermatology, University of Amsterdam, Meibergdreef 9, 1105 AZ, Amsterdam, The Netherlands; 2 Academic Medical Center, Department of Medical Psychology, University of Amsterdam, Meibergdreef 9, 1105 AZ, Amsterdam, The Netherlands; 3 Academic Medical Center, Department of Dermatology, University of Amsterdam, P.O. Box 22660, 1100 DD, Amsterdam, The Netherlands

**Keywords:** Dermatology, Randomized controlled trial, Patient-reported outcomes, Health-related quality of life, Intervention, Skindex-29, Psoriasis, Biologic, Etanercept

## Abstract

**Background:**

Interest in health-related quality of life (HRQoL) outcome research in dermatology is increasing, especially in the systemic treatment of psoriasis with biologic agents. In other specialties, such as oncology, the application of a HRQoL intervention is considered to be an aid for monitoring disease and treatment over time, for the communication with the patient, and for improving treatment outcome. However, in dermatology practice, the application of this intervention is relatively new. Moreover, evidence on the effectiveness of a HRQoL intervention in dermatology is missing. It is hypothesized that the application of a HRQoL intervention in dermatology practice will have a positive impact on patients’ HRQoL as well as on doctor-patient communication.

**Methods/design:**

In a prospective multicenter cluster randomized controlled trial, patients diagnosed with moderate to severe psoriasis who receive biologic treatment, will be followed for 48 weeks. The study sites, and not the patients, will be randomly allocated via a computer-based randomization system to either the intervention (treatment with etanercept and standardized HRQoL assessment and communication) or the control group (treatment with etanercept alone). The HRQoL intervention will include 1) the electronic assessment of the Skindex-29, a well-studied dermatology-specific HRQoL questionnaire, and 2) the communication of the resulting Skindex-29 data with the patient. Prior to study start, dermatologists in the intervention group will be educated and trained in standardized HRQoL assessment and communication using the Skindex-29. At six consecutive visits, patients at study sites in the intervention group will be asked to complete the Skindex-29 on a desk-top pc at the clinic, just before their consultation with the dermatologist. A print-out of the completed questionnaire will be made and, guided by this print-out, feedback on the HRQoL scores will be given during the consultation. Primary outcome parameters are the impact of the HRQoL intervention on patients’ HRQoL, and the effect of the HRQoL intervention on doctor-patient communication. Secondary outcomes include health status and disease severity.

**Trial registration:**

The Netherlands National Trial Register (NTR): NTR1364.

## Background

Patient-reported outcomes (PROs) are any aspect of a patient’s health status that comes directly from the patient without the interpretation of anyone other than the patient [1]. PROs provide information on the disease or treatment from a patient’s perspective. Examples of PROs are disease severity, general health status, adherence to treatment, satisfaction with treatment, and health-related quality of life (HRQoL). In accordance with the definition of the World Health Organization, HRQoL can be defined as a reflection of patients’ physical, psychological, and social functioning and well-being [2], and is generally considered as a key outcome parameter. Chronic skin diseases, such as psoriasis, are known to have a relatively high, negative impact on HRQoL [3]. As a result, interest in HRQoL outcome research in dermatology is increasing, especially in the systemic treatment of psoriasis with biologic agents.

Psoriasis is a systemic inflammatory skin disease with increased epidermal proliferation, affecting 2% of the population [4]. Common physical complaints of this chronic skin disease are skin soreness, burning sensations, itching, and joint pain. Therapies include topical treatments (for example, topical corticosteroids), photo(chemo)therapies (for example, UV-B and PUVA), and the conventional systemic treatments (for example, methotrexate and cyclosporin). For patients who fail or who are contraindicated or intolerant to these conventional treatments, biologic response modifiers, or biologics, are available, such as adalimumab, etanercept, infliximab, and ustekinumab. However, since dermatological treatment can only offer a temporary suppression or remission of symptoms, treatment efforts may increasingly be directed towards both a decrease of disease severity and an increase of a patient’s HRQoL.

Previous research, including studies in oncology, suggested that the application of a HRQoL intervention is considered to be an aid for monitoring disease and treatment over time, for the communication with the patient, and for improving treatment outcome [5,6]. However, in dermatology practice, the application of a HRQoL intervention is relatively new. Moreover, evidence on the effectiveness of a HRQoL intervention in dermatology is still missing. The present study may rectify this deficiency. It is hypothesized that a HRQoL intervention will significantly improve patients’ HRQoL, and will facilitate doctor-patient communication. In addition, this study offers the opportunity to examine the course of HRQoL during treatment, the mid- and long-term effects of treatment on HRQoL, and the relationship between disease severity and HRQoL during treatment.

## Methods/Design

### Design of the study

This prospective multicenter randomized controlled trial (RCT) will include a 48-week follow-up period with six consecutive, predefined visits: V1 (week 0; baseline visit), V2 (week 6; 6 weeks after the first injection of etanercept), V3 (week 12; 12 weeks after the first injection), and continuing on in this manner for V4 (week 24), V5 (week 36), and V6 (week 48). For a comprehensive study flow chart, see Additional file 1 (for the intervention group) and Additional file 2 (for the control group). Via ALEA, a software package to support online patient registration and randomization in healthcare research, the participating study sites will be cluster randomized [www.tenalea.com]. Thus, it is not the patients, but the study sites that will be randomly allocated to either the intervention group (treatment with etanercept and standardized HRQoL assessment and communication) or the control group (treatment with etanercept alone). With this computer-based system, allocation of concealment will be assured. Random assignment of patients instead of study sites was considered, but rejected due to practical limitations, that may cause a contamination effect.

**Figure 1:**
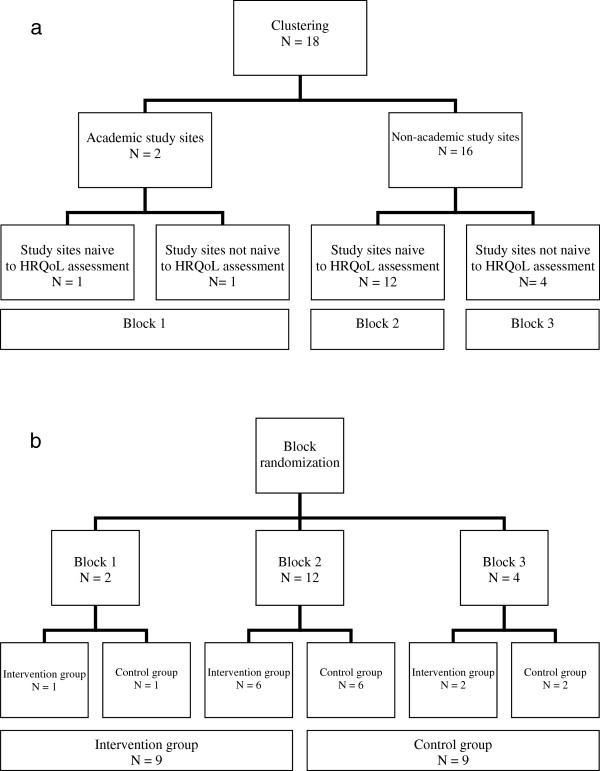
**Clustered block randomization (a).** Clustering of the participating study sites (*N*=18). (**b**). Block randomization of the clustered study sites (*N*=18).

In order to create equal comparison groups, the randomization will be stratified by four clusters: academic centers versus non-academic centers, and centers naive to HRQoL assessment and communication versus centers not (completely) naive (Figure 1a). Study sites that have participated in a national Working Group Quality of Life Assessment in Dermatological Practice (2007 to 2008) will be considered not naive, since these sites do have a high interest in, and are involved in HRQoL assessment at their local clinical settings. This will result in a randomization scheme consisting of three blocks (Figure 1b). Herewith, the sizes of the intervention and control group will be similar, taking an equal distribution of type of study sites into account.

Since the intervention concerns both patients (for example, completion of the intervention questionnaire) and dermatologists (for example, providing feedback on the HRQoL scores of the intervention questionnaire), dermatologists in the intervention group will be educated and trained in standardized HRQoL assessment and communication prior to study start, during the ‘Intervention Training’ session on group level. The aim of this training is to enable dermatologists in the intervention group to adequately discuss HRQoL scores, as well as aspects of HRQoL, such as coping behavior and disease management. As a result, dermatologists will become more familiar and, thereby, comfortable with HRQoL assessment and communication in clinical practice. To this aim, the Intervention Training will include evidence-based background information on the construct HRQoL, its relevance to clinical practice, and the impact of psoriasis on HRQoL [5-10]. Dermatologists will be trained in the electronic assessment of the intervention questionnaire, the Skindex-29, and the interpretation of its scores. In addition, information on coping behavior and disease management will be provided.

In this study design neither the patients nor the dermatologists can be blinded to the intervention.

### Setting

The study will be conducted at 18 outpatient dermatology clinics in the Netherlands. Psoriasis patients will be consecutively invited to participate in the study after a medical decision is made to start biologic treatment with etanercept. Patients eligible for the study are diagnosed with a moderate to severe psoriasis (that is, a Psoriasis Area and Severity Index (PASI) of ≥8), and are 18 years or older at the time of informed consent. Patients who are mentally and/or physically unable to complete the study questionnaire(s), who speak the Dutch language insufficiently to fully understand and complete the intervention and/or study questionnaire(s), or who are not willing or not able to discuss HRQoL-issues, will be excluded from participation. A patient information form will be handed to all potential participating patients. Written informed consent needs to be obtained from all participating patients prior to study start.

This study is registered in the Dutch Trial Register (NTR1364). The study protocol has been reviewed and approved by the Competent Authority of The Netherlands (Centrale Commissie Mensgebonden Onderzoek - CCMO) (NL24494.018.08 BI), and reviewed by the Medical Ethics Committee of the Academic Medical Center, University of Amsterdam (EC AMC) (MEC 08/302). The EC AMC exempted this study for ethical approval. A written confirmation of this statement was given by the EC AMC (Ref: MEC 08/302# 08.17.1716; MEC 08/302# 08.17.1933). The study will be conducted in accordance with the applicable laws and regulations following the Declaration of Helsinki protocols.

### Participants

**Figure 2:**
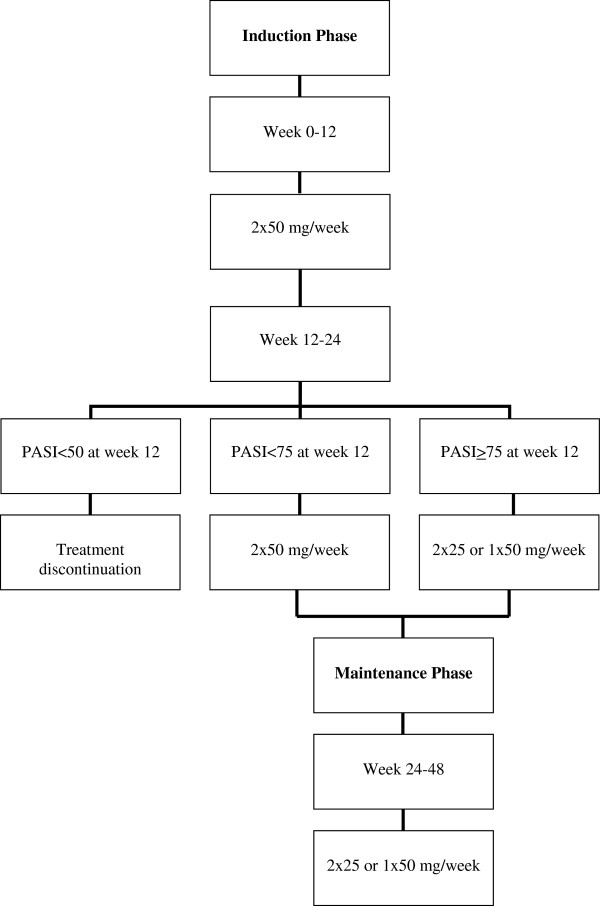
Per-protocol dose regimen schedule for etanercept in the treatment of moderate to severe psoriasis.

Psoriasis patients who will receive etanercept treatment by prescription from their dermatologists will be asked to participate in the study [11-14]. Thus, all included patients of both the intervention and control group will receive etanercept, as a constant factor, in accordance with routine clinical practice and, ideally, following the Dutch treatment guidelines for psoriasis: a PASI ≥8; ineffective or contra-indications to PUVA treatment twice weekly for 10 weeks; ineffective or contra-indications to treatment with cyclosporine 3-5 mg/kg/day for 16 weeks; and/or ineffective or contra-indications to treatment with methotrexate 22.5 mg/day for 16 weeks [15]. The per-protocol dose regimen of etanercept in week 0 to 12 (V1 to V3) is in accordance with the current Dutch summary of product characteristics (SmPC), namely: 2 x 50 mg/week. Depending on the PASI as measured at week 12 (V3), patients either discontinue treatment in case of PASI <50, or continue treatment during week 12 to 24 (V3 to V4). This treatment phase is called the induction phase. If PASI <75, patients will continue treatment with etanercept with 2 x 50 mg/week; if PASI ≥75, patients will continue with 2 x 25 or 1 x 50 mg/week. However, current clinical practice showed that a growing number of Dutch dermatologists continue treatment directly after the maximum treatment period of 24 weeks. Supporting evidence was found for an entire dosage regimen (week 0 to 48) in the British Association of Dermatologists Guidelines, in the German guidelines for systemic therapy, and in a study on the long-term safety and efficacy of 2 x 50 mg/week [16-19]. Per study protocol, patients will continue treatment during week 24 to 48 (V4 to V6), with 2 x 25 or 1 x 50 mg/week, which is called the maintenance phase (see Figure 2). The dosing schedule as suggested per protocol will be used as a guide for dermatologists. However, the actual dosing schedule to be given to patients will depend on routine clinical practice and the dermatologists’ prescriptions. Previous treatment with any biologic is allowed, taking the applicable wash-out period into account prior to treatment with etanercept. Concomitant topical treatment will be permitted.

### Intervention

The HRQoL intervention in this study consists of 1) the electronic assessment of the Skindex-29, a well-studied dermatology-specific HRQoL questionnaire, and 2) the communication of the resulting Skindex-29 data with the patient. The Skindex-29 consists of 29 questions, or items (for example items on pain, itch, embarrassment, frustration, social life, and interaction with others). Questions concern patients’ perception of the impact of the skin disease on aspects of HRQoL during the past week. These 29 items form three domains: symptoms, emotions, and functioning. Responses are given on a five-point response scale (ranging from ‘never’ to ‘all the time’), where scores are transformed to a 100-point scale, and domain and overall scores are being calculated by averaging responses to items in a given domain. Higher scores are indicating lower levels of HRQoL [20,21]. Several reviews suggested that the Skindex-29 is considered to be the instrument of choice in dermatology [9,22]

Before study start, dermatologists in the intervention group will be supported in the installation of the electronic version of the Skindex-29 on a desktop pc at the study sites, including a connection with a printer device. Prior to each consultation, patients in the intervention group will be asked to complete the Skindex-29 on a desk-top pc at the clinic. Patients will receive instructions from a dermatologist, or a designated representative, on how to complete this electronic questionnaire, without providing any information on the content or answers of the items. After completion, both the patient and the dermatologist will receive a print-out of the questionnaire, including the answers given and a matrix visually displaying the domain and overall scores. Guided by this print-out, the dermatologist will be able to systematically discuss the Skindex-29 answers and scores with the patient. In addition, and during consecutive consultations, the patient and the dermatologist will also be able to follow the HRQoL-scores over time as being displayed by this matrix showing repeated measurements.

The HRQoL intervention will be supported by patient information on quality-of-life, coping behavior, and disease management. At the first consultation, dermatologists will be instructed to provide two brochures to the patient; one with general information on HRQoL, and one on coping with and the management of psoriasis. At the second consultation, patients will receive another brochure about the treatment of psoriasis, including a chapter on HRQoL. In addition, a quality-of-life issue of a patient magazine from the Dutch Psoriasis Foundation will be provided to patients as well. At the third consultation, patients will receive a DVD on coping with psoriasis. At the fourth consultation, patients will receive a book entitled: ‘Psoriasis - Image, Experience, Treatment’, and patients will be instructed to read chapter 15: ‘The experience of psoriasis’. And, at the fifth consultation, patients will be asked to read chapter 16: ‘Coping with psoriasis’. Patients will be instructed to read and to watch the applicable patient information at home and prior to their subsequent consultation. The patient information can be stored in a study specific binder called ‘My Quality of Life’.

Additionally, all patients, both in the intervention and control group, will be asked to complete a diary to record the etanercept injections. These diaries will be used for monitoring the drug compliance.

### Comparison

Patients in the control group will not receive the HRQoL intervention, but will receive etanercept treatment only. The consultation will be conducted according to routine clinical practice, or usual care. Dermatologists in the control group will be asked to stay naive to HRQoL assessment and communication during the course of this study; that is, they are not to implement electronic HRQoL assessment with and communication about the Skindex-29 during their consultations with study participants.

#### Outcome measures

The primary outcome parameters in this study are 1) the impact of the HRQoL intervention on patients’ HRQoL and 2) the effect of the HRQoL intervention on doctor-patient communication. Since the Skindex-29 is used as the intervention questionnaire, the impact on HRQoL will be measured with another dermatology-specific HRQoL questionnaire, namely the Dermatology Life Quality Index (DLQI). The DLQI consists of ten items and each item is scored on a four-point scale ranging from ‘not at all/not relevant’ to ‘very much’. Scores of individual items (0 to 3) are added to yield a total score (0 to 30). Higher scores mean greater impairment of a patient’s HRQoL [23,24]. A study-specific, two-dimensional communication questionnaire will be used to measure the effect on doctor-patient communication. This communication questionnaire consists of two parts, measuring: 1) the satisfaction with doctor-patient communication and 2) the quantity of HRQoL communication during consultations. With approval from the authors, the first part of the communication questionnaire is a slightly adapted version of the Patient Satisfaction Questionnaire and includes items on satisfaction with information, (emotional) support, communication, and fulfillment of needs [25]. Answers are given on a visual analogue scale (VAS) ranging from 0 to 10, for example, ‘not at all satisfied’ to ‘very much satisfied’, or ‘not at all important’ to ‘very much important’. The cut-off point for satisfaction will be 6.0 (60%). The second part of the communication questionnaire includes items on symptoms, mood or emotions, social functioning, and overall quality-of-life. Patients will be asked if these aspects were discussed during the patient consultation or not, by ticking ‘yes’, ‘no’, or ‘I don’t know’. The communication questionnaire was successfully pilot tested in seven in-patients with psoriasis at the Academic Medical Center, Amsterdam.

The secondary efficacy endpoints include health status and disease severity. Health status will be measured with the Medical Outcomes Study 36-item Short-Form General Health Survey (SF-36), a well-established, eight-dimensional, generic HRQoL instrument [26,27]. The items represent issues relevant to health status, and relate to the past four weeks. The SF-36 will be completed at week 0 (V1), week 24 (V4), and week 48 (V6) by patients in both the intervention and control group. Disease severity will be measured with the PASI, the most widely used instrument for the measurement of the severity of psoriasis [28], and with an investigator global assessment (IGA) and a patient global assessment (PGA). The IGA is asking dermatologists: ‘In your opinion, how severe is your patient’s current skin condition?’, and the PGA is asking patients: ‘How severe is your current skin condition?’. Answers can be given on a five-point scale, ranging from ‘not severe’ to ‘very severe’. All patients will be asked to complete this set of questionnaires at the end of each patient consultation.

In addition, two evaluation questionnaires will be introduced to measure 1) aspects on satisfaction and 2) aspects on the feasibility of HRQoL assessment and communication in dermatology practice. First, at week 24 (V4) and week 48 (V6), an overall evaluation questionnaire will be handed to dermatologists in the intervention group, and to patients in both the intervention and the control group. This questionnaire comprises a study-specific questionnaire about 1) satisfaction with the treatment process, including doctor-patient communication, and 2) satisfaction with treatment outcomes. For example, ‘How satisfied are you about the conversations you have had with your doctor/patient?’. Answers are given on a VAS, ranging from ‘not at all satisfied’ to ‘very much satisfied’. Second, after each patient consultation, dermatologists in the intervention group will be asked to answer questions on aspects of feasibility, such as completion time, duration of the consultations, and (the number of) aspects of HRQoL discussed during the consultations. Dermatologist in the control group will only be asked about the duration of the consultations.

#### Data collection

A Case Report Form (CRF) will be provided for each patient. All protocol-required information that needs to be collected during this study will be entered by the dermatologist, or a designated representative. Baseline characteristics will be obtained at visit 1 (week 0).

#### Safety

All patients will be monitored for safety during their participation in the study, including adverse events (AEs) and premature discontinuation from the study. AEs are defined as any undesirable experience occurring to a patient during the study, whether or not considered related to etanercept. Serious adverse events (SAEs) will be recorded by the responsible dermatologist and reported within 24 hours of notification to the trade holder of etanercept. According to the Dutch Personal Data Protection Act, all data will be handled confidentially and anonymously, and will be stored for 15 years after study close out (that is, last patient last visit).

### Analysis

#### Power calculation

The sample size calculation is based on the two primary outcome parameters and its corresponding outcomes measures, namely the DLQI and the communication questionnaire.

Based on previous research, we will expect a HRQoL improvement, as measured with the DLQI, of 1.0 standard deviation (SD) in the intervention group and of 0.7 in the control group. We expect a communication effect size of 0.8 (that is, an improvement of 0.8 SD) in the intervention group, and of 0.4 in the control group. With 100 patients in each group, the power to detect a longitudinal effect would be larger than 99%, assuming a significance level of 5% and an autocorrelation (that is, the correlation between scores at baseline and end of induction phase) of 0.5.

The power to detect differences in longitudinal DLQI effects between the intervention and control group will be 56%. In case of one-tailed testing, the power to detect differences in longitudinal DLQI effects between the intervention and control group will be 68%. A mean DLQI score of 12.0 (SD 7.0), and a clinically meaningful improvement of 5 points is expected. In the intervention group, an additional improvement of 2 points (effect size: 0.3) is expected.

The power to detect differences in longitudinal communication effects between the intervention and control group is 80%. The power calculation is conservatively based on an estimate of 200 patients; that is, it is based on 100 patients per group and an expected withdrawal rate of approximately 20%.

#### Statistical analysis

Statistical analyses will comprise two components: 1) the induction phase analysis (week 0 to week 24) and 2) the maintenance phase analysis (week 24 to 48).

The induction phase analysis will concern all patients who receive any treatment with etanercept, who respond to the study questionnaires on HRQoL, communication, health status, and disease severity, and of whom PASI is assessed at baseline. The last visit of patients who may discontinue from treatment for any reason, including patients who will not meet the criteria to continue treatment with etanercept after week 12 (PASI <50), will be considered as End of Induction Phase (week 24).

The maintenance phase analysis will concern all patients who receive any treatment with etanercept after week 24, who respond to the questionnaires, and of whom PASI is assessed at week 24. The last visit of patients, who may discontinue from treatment for any reason, will be considered as End of Maintenance Phase as well as End of Treatment (week 48).

All analyses will be performed on an intention-to-treat basis. In any case where patients prematurely discontinue from treatment, they will be asked to complete an Early Termination visit. The content of this visit is similar to V6, and data will be handled based on the ‘last value carried forward’ principle.

Patient characteristics will be described by randomized groups. Categorical data will be summarized as frequencies and percentages. Continuous data will be summarized by means and standard deviations. Linear mixed model analysis will be used to investigate the course of the outcomes (that is, DLQI scores for HRQoL; communication scores for doctor-patient communication; SF-36 scores for health status; and PASI, IGA, and PGA for disease severity), and to test the effects of possible explanatory variables on the outcome variables. Longitudinal models will have a random intercept to account for individual differences at baseline, fixed regression coefficients for each of the measurement occasions following baseline, for the intervention effect, for study sites (to account for differences between sites), and for possible other explanatory variables (such as age and sex). We will investigate which longitudinal structure is most appropriate, whether one or more of the fixed regression coefficients should be considered random, and whether there are interaction effects.

## Discussion

This study investigates the impact of HRQoL assessment and communication on patients’ HRQoL, and its effect on doctor-patient communication. To the best of our knowledge, this is the first RCT that examines the effectiveness of such HRQoL intervention in patients with moderate to severe psoriasis.

Several studies in, for example, oncology showed a positive impact on patients’ well-being as well as a significant increase in the discussion of symptoms. Valderas and colleagues (2008) [29] conducted a systematic review on the impact of PRO assessment in clinical practice in different clinical settings, such as internal medicine, oncology, and primary care. They concluded that, because the studies analyzed were heterogeneous in the types of setting, patients, intensity of intervention, and diversity of outcomes, no apparent conclusion could be drawn. In addition, the included studies were of limited methodological quality. Despite this, they found grounds for optimism, and they recommended well-designed and well-conducted future randomized studies [29].

A major strength of this study is that clinical staff, rather than research staff, is responsible for the implementation of the intervention. Herewith, the HRQoL intervention is implemented in a regular clinical setting. In addition, the HRQoL intervention will be evaluated in multiple clinical settings with diverse doctor and patient samples.

A few limitations of this study need to be addressed. First, our patient sample cannot be considered truly representative for psoriasis patients in general since only psoriasis patients who are being treated with etanercept will be asked to participate in the study. This may affect the external validity. Treatment with other biologics was considered, but rejected due to methodological limitations, such as route of administration, dosing schedule, and corresponding consultations to the dermatologists, that may affect the outcome. Second, the improvement of patients’ HRQoL over time may be due not only to the intervention, but also to their psoriasis treatment with a biologic [30,31]. However, an additional improvement on top in HRQoL is expected because of the HRQoL intervention. Third, neither the dermatologists nor the patients can be blinded in this study design, which may affect the internal validity. And lastly, we used block randomization to generate equal comparison groups with respect to academic versus non-academic centers and centers being naive versus not-naive to HRQoL assessment. However, since only two academic centers agreed to participate, with one center being naive and the other not naive to HRQoL assessment, the intervention and control conditions in the academic centers are not comparable with respect to this latter variable.

The findings to be reported in this study are the first in dermatology. Therefore, some caution is needed when interpreting the results of this study, and confirmation through future study results is necessary. Nevertheless, the results of this study may be encouraging for further research and future use in dermatology practice.

## Trial status

Patient recruitment is ongoing.

## Abbreviations

AE: Adverse event; CRF: Case report form; DLQI: Dermatology Life Quality Index; EC AMC: Ethics Committee Academic Medical Center; HRQoL: Health-related quality of life; IGA: Investigator global assessment; PASI: Psoriasis Area and Severity Index; PGA: Patient global assessment; PROs: Patient-reported outcomes; RCT: Randomized controlled trial; SAE: Serious adverse event; SD: Standard deviation; SF-36: Medical Outcomes Study 36-item Short-Form General Health Survey; SmPC: Summary of product characteristics; VAS: Visual analogue scale.

## Competing interests

The authors declare that they have no competing interests.

## Authors’ contributions

JdK is the principle investigator. CP is the coordinating investigator. JdK and CP are responsible for the design of the study and the study protocol. PS, MS, and MdR have contributed to the design of the study and to the content of the study protocol with important intellectual revisions. CL has critically reviewed the content of the study protocol. CP is responsible for drafting the protocol manuscript. All authors have read and approved the final protocol manuscript.

## Supplementary Material

Additional file 1 Study flow chart intervention group. Click here for file

Additional file 2 Study flow chart control group. Click here for file
