# Supplementary material for: The efficacy of a health-related quality-of-life intervention during 48 weeks of biologic treatment of patients with moderate to severe psoriasis: study protocol for a multicenter randomized controlled trial
Source: Trials. 2012 Dec 8;13:236. doi: 10.1186/1745-6215-13-236 (PMC3560082; doi:10.1186/1745-6215-13-236)
Supplement: Additional file 1 — Study flow chart intervention group. [file 1745-6215-13-236-S1.doc]

**Additional file 1a. Study flow chart intervention group**

| **VISIT 🡪** | **1** | **2** | **3** | **4** | **5** | **6** |  |  |
| --- | --- | --- | --- | --- | --- | --- | --- | --- |
| **WEEK 🡪**  **🡫 STUDY PART** | **Wk 0**  **Baseline** | **Wk 6** | **Wk 12** | **Wk 24** | **Wk 36** | **Wk 48 EOS¹** | **EV²** | **ET³** |
| **In- and exclusion criteria** | X |  |  |  |  |  |  |  |
| **Informed Consent** | X |  |  |  |  |  |  |  |
| **Demographics** | X |  |  |  |  |  |  |  |
| **Medical history** | X |  |  |  |  |  |  |  |
| **Medical status** |  | X | X | X | X | X | X | X |
| **Medication** | X | X | X | X | X | X | X | X |
| **Adverse Events (AEs)** |  | X | X | X | X | X | X | X |
| **Skindex-29** | X | X | X | X | X | X | X | X |
| **Communication Questionnaire** | X | X | X | X | X | X | X | X |
| **DLQI** | X | X | X | X | X | X | X | X |
| **SF-36** | X |  |  | X |  | X |  | X |
| **PASI** | X | X | X | X | X | X | X | X |
| **Global Disease Severity** | X | X | X | X | X | X | X | X |
| **Overall Evaluation** |  |  |  | X |  | X |  | X |
| **Experience with the intervention questionnaire** | X | X | X | X | X | X | X | X |
| **Checklist** | X | X | X | X | X | X | X | X |

¹ End Of Study visit

² Extra visit, if applicable

³ Early Termination visit, if applicable
